# Changing household dietary behaviours through community-based networks: A pragmatic cluster randomized controlled trial in rural Kerala, India

**DOI:** 10.1371/journal.pone.0201877

**Published:** 2018-08-22

**Authors:** Meena Daivadanam, Rolf Wahlström, T. K. Sundari Ravindran, P. Sankara Sarma, S. Sivasankaran, K. R. Thankappan

**Affiliations:** 1 Department of Food, Nutrition and Dietetics, Uppsala University, Uppsala, Sweden; 2 Department of Public Health Sciences (Global Health), Karolinska Institutet, Stockholm, Sweden; 3 Achutha Menon Centre for Health Science Studies, Sree Chitra Tirunal Institute for Medical Sciences and Technology, Trivandrum, India; 4 Department of Public Health and Caring Sciences, Uppsala University, Uppsala, Sweden; 5 Department of Cardiology, Sree Chitra Tirunal Institute for Medical Sciences and Technology, Trivandrum, India; Leibniz Institute for Prevention Research and Epidemiology BIPS, GERMANY

## Abstract

**Trial design:**

With the rise in prevalence of non-communicable diseases in India and Kerala in particular, efforts to develop lifestyle interventions have increased. However, contextualised interventions are limited. We developed and implemented contextualised behavioural intervention strategies focusing on household dietary behaviours in selected rural areas in Kerala and conducted a community-based pragmatic cluster randomized controlled trial to assess its effectiveness to increase the intake of fruits and vegetables at individual level, and the procurement of fruits and vegetables at the household level and reduce the consumption of salt, sugar and oil at the household level.

**Methods:**

Six out of 22 administrative units in the northern part of Thiruvananthapuram district of Kerala state were selected as geographic boundaries and randomized to either intervention or control arms. Stratified sampling was carried out and 30 clusters comprising 6–11 households were selected in each arm. A cluster was defined as a neighbourhood group functioning in rural areas under a state-sponsored community-based network (*Kudumbasree*). We screened 1237 households and recruited 479 (intervention: 240; control: 239) households and individuals (male or female aged 25–45 years) across the 60 clusters. 471 households and individuals completed the intervention and end-line survey and one was excluded due to pregnancy. Interventions were delivered for a period of one-year at household level at 0, 6, and 12 months, including counselling sessions, telephonic reminders, home visits and general awareness sessions through the respective neighbourhood groups in the intervention arm. Households in the control arm received general dietary information leaflets. Data from 478 households (239 in each arm) were included in the intention-to-treat analysis, with the household as the unit of analysis.

**Results:**

There was significant, modest increase in fruit intake from baseline in the intervention arm (12.5%); but no significant impact of the intervention on vegetable intake over the control arm. There was a significant increase in vegetable procurement in the intervention arm compared to the control arm with the actual effect size showing an overall increase by19%; 34% of all households in the intervention arm had increased their procurement by at least 20%, compared to 17% in the control arm. Monthly household consumption of salt, sugar and oil was greatly reduced in the intervention arm compared to the control arm with the actual effect sizes showing an overall reduction by 45%, 40% and 48% respectively.

**Conclusions:**

The intervention enabled significant reduction in salt, sugar and oil consumption and improvement in fruit and vegetable procurement at the household level in the intervention arm. However, there was a disconnect between the demonstrated increase in FV procurement and the lack of increase in FV intake. We need to explore fruit and vegetable intake behaviour further to identify strategies or components that would have made a difference. We can take forward the lessons learned from this study to improve our understanding of human dietary behaviour and how that can be changed to improve health within this context.

## Introduction

### Background

The phenomenon of nutrition transition has been studied extensively on both global and regional scale, and its temporal link with emerging diet-related non-communicable diseases (NCDs) such as diabetes, hypertension, and cardiovascular diseases has been widely established [1]. The most consistent linkages have been demonstrated in terms of total calories consumed and the content of foods (specifically, in terms of their fibre, salt, sugar or fat content) [1]. This forms the evidence base for all current NCD prevention efforts involving diet. World Health Organization (WHO) has recommended a daily intake of five servings of fruits and vegetables (FV) and a restricted use of sugar, salt and oil [2]. The recognition that changing or maintaining healthy dietary behaviours is a complex process has resulted in the routine use of well-established behavioural strategies in dietary interventions [3]. This process includes decision-making mechanisms that involve other cultural and contextual factors [4], not directly related to the nutritive value of foods.

#### Food decision-making dynamics

Decision-making related to food has been studied predominantly from marketing, sociological or economic perspectives, rather than from the health perspective [5–12]. In spite of the differing nature of the societies, where these studies have been carried out, there are certain common factors. In consumer research, food is a commodity that is consumed jointly by members of a household; and greater family influence has been hypothesized for such commodities [5]. Hence, irrespective of culture, food choices involve a greater collective component. Understanding the dietary decision-making dynamics, common and context-specific factors, are of importance to develop appropriate intervention strategies.

Only a few studies from India have looked at decision-making in households (mainly from a consumer perspective with food as one of the commodities); or the implications of bargaining power in terms of gender-power relations [6, 7, 9]. There have also been attempts to understand food habits and choices among different population groups [13, 14], though not from the perspective of diet and health. The prevalence of NCDs varies widely between the different states in India; and Kerala state owing to its advanced stage of demographic and epidemiologic transition ranks the highest. For example, the age-adjusted prevalence of type 2 diabetes for all of India is 8%, whereas the state average for Kerala is 14.3% among men and 17.8% among women. In fact, the prevalence is highest among rural women in Kerala (22.2%) [15, 16], making it relevant and urgent to address primary prevention of NCDs in rural Kerala. This in turn calls for more focused attention on household food behaviours in order to gain a deeper understanding of the process and to develop and implement contextualised interventions.

#### Dietary interventions in the Indian context

Most of the initial NCD intervention studies conducted among the adult population in India have focused on diabetes and included diet as a component of lifestyle modification in a clinic or community setting [17, 18]. A community-based intervention for NCD risk factor reduction with an overall lifestyle modification program–multiple strategies and activities covering individual and community empowerment, advocacy and reorientation of health services–demonstrated only a modest, though statistically significant increase in the proportion of individuals consuming more than five servings per day of fruits and vegetables in spite of the very low baseline levels (women: from 3% at baseline to 5%, and men: from 5% at baseline to 9%) [19]. Other intervention studies that have a dietary component have not yet published their results [20, 21].

The dietary component in these interventions has been restricted to counselling by nutritionists based on the National Institute of Nutrition’s dietary guidelines for Indians [22]; and the cultural adaptation has been limited to inclusion of local foods in the menu [17, 19]. A couple of studies have included culturally appropriate dietary education; addressed cultural barriers to dietary change [18, 21]; used local facilitators to deliver life-style classes [21]; promoted local low-cost resources; and used cooking demonstrations, recipe competitions and model meals [18]. Food preparation is an expected and central activity in Indian households, intrinsically linked to a woman’s identity [13]. Dietary interventions in India should take into account these family dynamics, the intrinsic roles of both genders, and the various societal and familial power relations [23]. There is still a gap in our basic knowledge related to dietary practices, perceptions and beliefs within communities. In addition, the most important source of dietary components in Indian households, especially in rural areas is still home-cooked food as opposed to processed food in more affluent countries. Dietary interventions in India need to integrate these realities with community-based approaches that modify existing dietary practices as a possible solution.

### Rationale

Considering the knowledge-practice gap related to dietary behaviour change interventions for the prevention of NCDs in the Indian context, we identified the need to develop, implement and test dietary behaviour change strategies informed by theory and in-depth formative research, focusing on households. The objective of this paper is to report the results of a pragmatic cluster randomized controlled trial (cRCT) developed and implemented as part of the Behavioural Intervention for Diet (BID) study [24] to assess the effectiveness of a contextualized and community-based approach utilizing sequential stage-matched intervention strategies [24–26] to change dietary behaviour in Kerala in terms of increased procurement and intake of fruits and vegetables, and reduced consumption of salt, sugar and oil.

## Methods

The trial details and results are reported here in accordance with the CONSORT guidelines [27]. Appropriate modifications were made based on CONSORT extensions for cluster trials and pragmatic trials [28, 29]. The study protocol paper with a detailed description of the study design and methodology has been published elsewhere in *Glob Health Action* 2013;6: 20993. (http://www.tandfonline.com/doi/full/10.3402/gha.v6i0.20993) [24].

### Objectives

**Primary objective**

- To test the effectiveness of a sequential stage-matched intervention strategy to increase the daily intake of fruits and vegetables (FV) defined as five servings–two fruit + three vegetable servings–by an absolute 20% from baseline in the intervention arm over a one-year intervention period.

**Secondary objectives**

- To increase household procurement of FV by a minimum of 20%, from baseline in the intervention arm over a one-year intervention period.
- To decrease household consumption of salt, sugar, and oil (SSO) by a minimum of 10%, from baseline in the intervention arm over a one-year intervention period.

### Study setting

Thiruvananthapuram (Trivandrum) district with a population of about 3.3 million inhabitants was chosen for two reasons: its human development index was similar to that of Kerala state, making it fairly representative of the state [16]; and its proximity to a premier public health institution in the country for ease of monitoring. Chirayinkeezhu taluk is one of the four revenue divisions of Thiruvananthapuram district with a population of 550 thousand (about 130 thousand households). It is divided into four block panchayats, which in turn consist of 22 *grama panchayats* (rural administrative unit) and two municipality areas (urban administrative unit). Each *grama panchayat* is further divided into 10–17 smaller administrative areas called wards.

### Study design and sampling procedure

**Fig 1:**
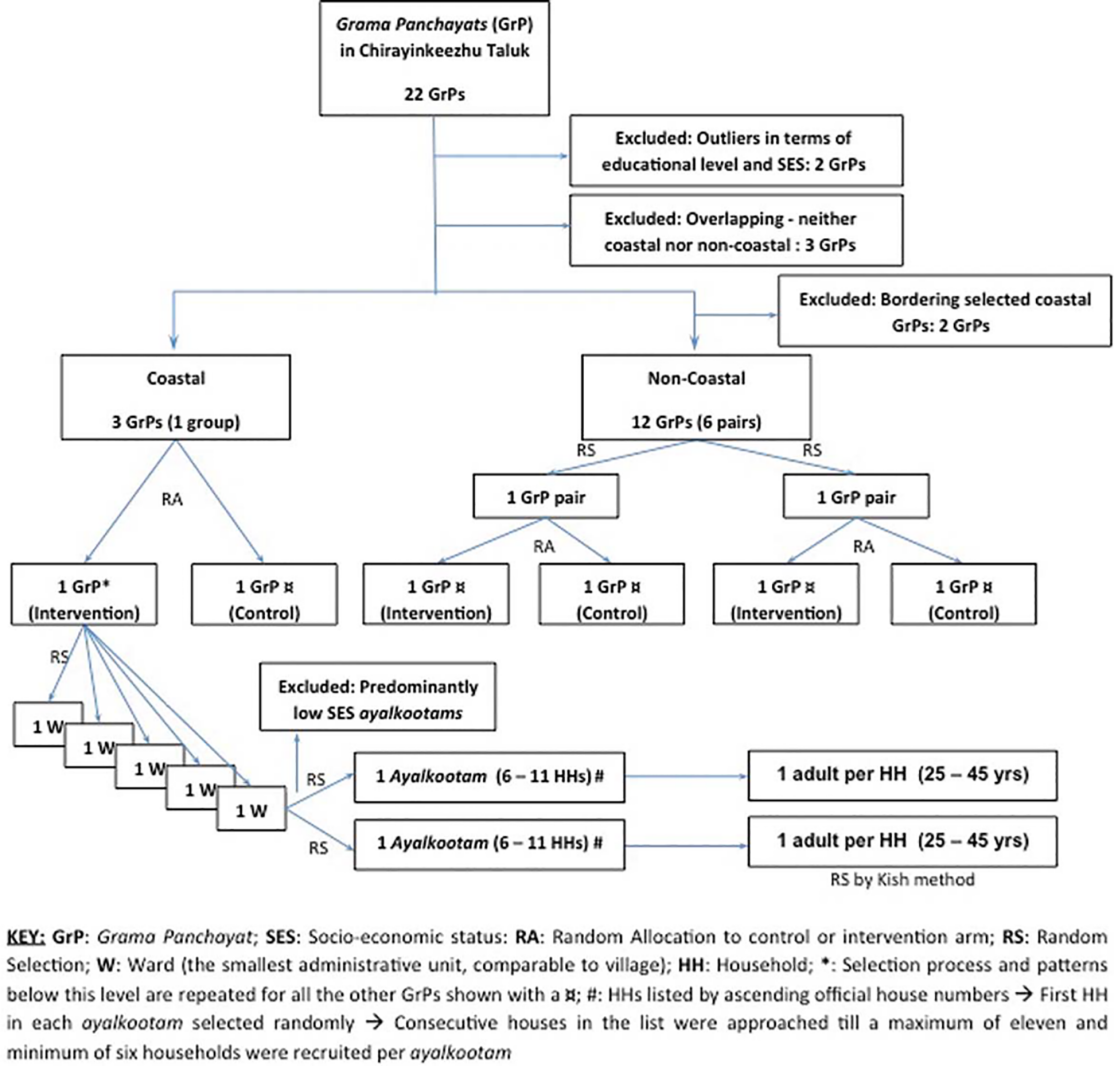
Randomization and selection for the cRCT from panchayat to individual levels. (Source: Daivadanam et al. Glob Health Action 2013, 6: 20993, http://dx.doi.org/10.3402/gha.v6i0.20993).

We used a cluster RCT design with two arms and fixed number of clusters (30 in each arm) and each cluster comprising 6 to 11 households (Fig 1). Multistage stratified cluster random sampling was carried out as follows and all random selections and allocations were done using an online random number generator [30] by two of the authors (MD and RW):

#### Stage 1: Sampling at the level of the grama panchayats

Six *grama panchayats* were selected out of 22 in the district (three each in the intervention and control arms) (Fig 1). *Grama panchayats* were paired to ensure an even distribution of key population characteristics and confounders between the control and intervention arms as follows: 1) coastal and non-coastal areas in separate pairs or groups; 2) proportion of middle-income households (HHs); and 3) no geographical continuity between panchayats belonging to one pair or group to prevent spill-over effect. One coastal group with three grama panchayats and two non-coastal pairs were identified after this process. In the coastal group, two were randomly selected of which the first was allocated to the intervention arm and the second to the control arm. In the non-coastal pairs, one panchayat in each pair was randomly allocated to the intervention arm and the remaining to the control arm.

#### Stage 2: Sampling at the level of the ward and ayalkootam

Below the level of the *grama panchayats*, five wards per panchayat and two *ayalkootams* per ward were randomly selected, before reaching the household level. Rural Kerala has neighbourhood groups organized through the *Kudumbasree mission* (women oriented, community based, and organized by the State Poverty Eradication Mission of Government of Kerala) known as *ayalkootams*. These are present in each ward and composed of women members from the community. Approximately 80% of low and middle-income HHs (at least one person from each HH) are registered members in these units. The clusters in this study were defined as the *ayalkootams*. *Ayalkootams* with predominantly low-income households (verified through *Kudumbasree* registers) were excluded, prior to the random selection of clusters.

#### Stage 3: Sampling at the level of the household

All HHs falling within geographic limits of the selected *ayalkoottams* were listed and screened before inclusion and exclusion criteria were applied. A HH was found to be eligible for recruitment into the study if it fulfilled all of the following inclusion criteria:

1. Belonged to the middle SES.
2. Had adult males or females 25–45 years of age.
3. Had resided in selected administrative areas (panchayats) of rural Chirayinkeezhu taluk for at least 6 months prior to the screening survey.

The socioeconomic status (SES) of HHs were identified for 2011 based on HH monthly per capita expenditure using the 2004–2005 poverty line [31] and the corresponding affluence line [32] for rural Kerala, after adjusting for inflation. The age group 25–45 years was targeted due to the high prevalence of migration in the younger age group and of NCDs among the older age group. Selection of individual participants was stratified by gender. One male or female individual within the prescribed age group was selected in each HH after exclusions based on the following criteria:

1. Took two or more meals away from home on most weekdays;
2. Had definite plans of migration during the study period;
3. Was pregnant; or
4. Had self-reported diabetes, hypertension, deranged lipids on medical treatment, and mental or other serious illness.

If more than one individual fulfilled none of the exclusion criteria, then Kish methodology was used [33]. The detailed design and methodology of the cRCT has also been published elsewhere [24].

### Sample size

Assuming an existing prevalence of 40% appropriate daily intake of FV (five servings) [34] and a 20% improvement in the intervention arm, with a power of 80% and an alpha error of 0.05, the minimum sample size required to compare two groups was calculated to be 98 in each group [35]. As intra-cluster correlation coefficient was not available for the outcome of interest, we incorporated a design effect of two. Hence, the minimum required sample size was 400 (200 intervention and 200 control), allowing for an attrition of 20%. Taking into consideration the risk factor prevalence up to 50% [16] and proportion of individuals in the required age group of 25–45 years, we screened 1237 households to recruit 479 individuals (intervention: 240; control: 239).

### Implications of formative research

Prior to the implementation of the trial, formative research was carried out to inform the development of contextualized intervention strategies:

#### 1) Needs assessment

A key finding of a previous study in Kerala was the collective nature of society and participants’ needs to include household and other community members, such as neighbours in intervention efforts [15].

#### 2) Dynamics of dietary decision-making in households

Our formative research [36] identified a delicate balance between the expectations, preferences and priorities of different household members and the many limitations that households face on a daily basis such as lack of resources or poor physical or financial access. Female heads of the households bear the disproportionate burden of maintaining this balancing act, due to their deep understanding of their respective households. Female heads of the households were also primarily responsible for food-related matters in the household and had the most knowledge about food procurement, preparation and consumption practices in the household. They were therefore found to be good proxies for their households with reference to diet-related matters [36].

#### 3) Conceptual model for household level dietary behaviour change

Informed by constructs from three health behaviour theories (trans-theoretical model or stages of change, the Health Belief Model and the Theory of Planned Behaviour), we constructed a conceptual model [26], which was contextualized through our own primary qualitative data. A matrix of behaviour change objectives and corresponding strategies and activities was developed at three levels: individual, household and community. The final components of the conceptual model were converted to either intervention strategies or key implementation elements for the cRCT [24].

#### 4) Household staging tool to assess readiness to change dietary behaviour

As there was no tool till date to assess the stage of change at a household level as opposed to an individual level; key findings related to food decisions [36] and the conceptual model [26] were used to develop such a tool that could be administered by lay community volunteers. This staging tool used a simple algorithm to compute the household stage based on responses from the female heads of households [37] and, consequently, the tool was administered only to the female heads of the households in this pragmatic RCT. Efficacy and perceived cooperation at the household level were found to be crucial identifiers when assessing the household’s readiness to change dietary behaviour. A pilot validation of the tool was carried out before its use in the cRCT [37].

### Description of the intervention

#### Intervention arm

Tables 1 and 2 list the intervention strategies and their mode of delivery, time-point of delivery and personnel responsible for the delivery of each strategy. A detailed study plan and description of intervention components and behaviour change strategies have been described elsewhere [24]. Intervention strategies at the individual level were restricted to participation in an initial face-to-face counselling and the telephone reminders. All other strategies were delivered at household or community (*ayalkootam)* level. The strategies were applied in a staggered manner in the intervention areas. The staging of households and delivery of corresponding strategies occurred at months 0 and 6. The intervention strategies were stage-matched and delivered as follows:

1. Pre-contemplation stage: only general strategies, i-iv (Table 1)
2. Intention stage: general strategies, i-iv (Table 1) + stage-matched strategies, a-c (Table 2)
3. Action stage: general strategies, i-iv (Table 1) + stage-matched strategies, a-e (Table 2)

**Table 1:** General intervention strategies (i-iv): Mode, time-points and personnel responsible for delivery.

S.No	Intervention strategy & description*	Mode of delivery	Time-point of delivery	Responsible personnel
**I**	*Counselling accompanied by home-visit*:Counseling using brief intervention format with female head of the household and selected adult in each household. Also open to other members of the household.	Face-to-face using pre-defined format	Month 1	Counselors–psychology students trained in brief intervention
		Telephone using pre-defined format	Month 6	Counselors–psychology students trained in brief intervention
		Home visit: face-to-face	Months 1 & 6 (if there is a change of stage)	Community volunteers trained to deliver specific strategies
**Ii**	*Reminder*:Reminder call to female head of the household.	Telephone using pre-defined format	Months 3 & 9	Community volunteers / study coordinator
		Face-to-face	Month 3 & 9	Study coordinator / community volunteer (if no response to telephone reminder calls)
**Iii**	*Awareness sessions*:General awareness sessions for community members. Also open to members from households not participating in the study.	Three group sessions for adults in each *ayalkootam* using flip charts	1) Months 2–3: Diet and NCDs 2) Months 4–5: Colour way to health 3) Months 9–10: Let us protect our family’s health	Sessions organized by community volunteers and conducted by psychology students trained with respect to materials and content
		Two group sessions for children in each *ayalkootam* using card games	Months 3–5: Diet and NCDs–concept of *plus* and *minus* foods Months 8–9: Colour way to health	Sessions organized by lay community volunteers and conducted by psychology students trained with respect to materials and content
**Iv**	*Sequential stage-matching*:Staging to differentiate households based on readiness to change dietary behavior in order to determine the stage-matched strategies to be delivered to each household	Household staging tool developed as part of formative research [36]	Month 0 & 6	Community volunteers trained to use the tool

*Additional details on intervention strategies have been published elsewhere [Glob Health Action 2013, 6: 20993, http://dx.doi.org/10.3402/gha.v6i0.20993].

**Table 2:** Stage-matched intervention strategies (a-e): Mode, time-points and personnel responsible for delivery.

S.No	Intervention strategy & description*	Mode of delivery	Time-point of delivery	Responsible personnel
**A**	*Easy formula*: Local measures and information on recommended levels of intake for fruits and vegetables, free sugars, salt and coconut and other cooking fat tailored for household size	Measurement kit and information booklet provided to each household—explained with practical examples during home visit	Month 1 and or 6 based on assessed stage of change of the household	Community volunteers trained to deliver intervention strategies
**B**	*Substitution*: 1) Fried snacks with fruits; 2) ready-to- eat or fried snacks with home-cooked or steamed snacks respectively; and 3) Colour of the lunch plate by increasing the portion of raw or cooked vegetables	Highlighting three strategies identified through formative research and outlined in information booklet—explained with practical examples during home visit	Month 1 and or 6 based on assessed stage of change of the household	Community volunteers trained to deliver intervention strategies
**C**	*Visibility*: Increase the display of FV on the dining table or easily accessible locations in the house and decreasing the display and access to jams, pickles, and fried snacks	Use of fruit basket provided in the household kit or removal of food items (high in salt, sugar and fat) from easily accessible locations—explained with practical examples during home visit	Month 1 and or 6 based on assessed stage of change of the household	Community volunteers trained to deliver intervention strategies
**D**	*Local varieties*: Encouraging the use of locally available FV by identifying nutritious local FV	Enable household members to identify and record local varieties with the help of the information booklet—explained with practical examples during home visit	Month 1 and or 6 during home visit based on assessed stage of change of the household	Community volunteers trained to deliver intervention strategies
**E**	*Budget re-allocation*: Enabling households to reallocate their budgets and increase the resources for FV	Using note pages in the information booklet to identify avoidable expenses—explained with practical example during home visit	Month 1 and or 6 based on assessed stage of change of the household	Community volunteers trained to deliver intervention strategies

*Additional details on intervention strategies have been published elsewhere [Glob Health Action 2013, 6: 20993, http://dx.doi.org/10.3402/gha.v6i0.20993].

The delivery of the general strategies was spread over nine months of the intervention period. Attendance to general awareness sessions were not mandatory in the protocol, however all participating households were encouraged to attend at least one awareness session for adults and one for children aged 6–15 years. All awareness sessions were open to adults and children in the community and were conducted within each of the communities represented by the selected *ayalkootams*. All activities described in Table 2 were delivered at the home of the participants. Further details on the methodology has been published elsewhere [24].

#### Control arm

The control areas received minimal intervention in the form of information on recommended levels of intake of the five dietary components, and general dietary information leaflets at the end of the baseline survey.

#### Tools

The following tools were developed and used in the intervention:

1. General dietary information leaflets for households in both intervention and control arms;
2. BID (Behavioural Intervention for Diet) Information booklet;
3. Household measurement kit: locally available serving spoon measuring one serving of cooked vegetables; oil jar (1L); calibrated cup to measure oil used for daily cooking; two containers with teaspoon measuring 5–6 grams of salt or sugar; and a fruit basket;
4. Three flip charts on each of the three themes of the awareness sessions for adults;
5. Two card games for children on the concept of ‘plus’ (healthy) and ‘minus’ (unhealthy) foods; and on ‘colours of health’. Games focusing on children were included based on findings from our formative research, that children greatly influenced HH food behaviours [36].

Details about the intervention tools have been published elsewhere [24]. One of the consistent messages was delivered throughout the intervention, during the community awareness sessions, counselling sessions and house visits, focused on making slow and steady changes to ensure the sustainability of any behaviour change in the long run.

#### Process

All face-to-face intervention activities took place at the homes of the participants or as per their convenience. All awareness sessions for adults were conducted either in one of the homes in the neighborhood or a nearby school or local administrative building. All awareness sessions for children were conducted in a nearby school or in one of the homes in the neighborhood.

### Data collection

#### Tools

Tools were developed for: 1) screening; 2) staging of households’ readiness-to-change [37]; 3) general questionnaire for baseline and end-line data collection at the individual and household levels, which included two 24-hour recalls (one weekend + one weekday recall using multiple pass method to measure FV intake for the selected individual); a structured section to collect data on reported monthly SSO utlilisation at the household level and a 2-week FV procurement diary for the household; 4) process documentation to keep track of the various processes and strategies involved in the intervention; and 5) template counselling sheets specific to stage of change of the household to guide counsellors during face-to-face and telephonic counselling and briefly document the content of the session. All data collection tools were translated to the local language Malayalam, back translated and pre-tested before the baseline data collection.

Data were collected from each household in three sittings:

1. General questionnaire for household and individual data + first 24-h recall (either weekend or weekday) + handing out the 2-week FV procurement diary with instructions and marked start and end dates;
2. second 24-hour recall (weekend or weekday based on first 24-h recall) + collection of the 2-week FV procurement diary;
3. extra visit as needed to complete any missing or questionable data,

All data related to the household including the 2-week FV procurement diary and the monthly SSO utlisation in the household were collected from the female head of the household, while 24-hour dietary recall was collected from the selected adult in each household. As the selection of the adult participant was done randomly using Kish methodology, in most cases, the female head of the household and the selected adult would not be the same individual. Further details about the data collection tools have been published elsewhere as part of the design and methodology of the study [24].

#### Process

The screening and recruitment of households and simultaneous baseline data collection took place from July to December 2011. Each participating household completed 12 months of intervention following which end-line data was collected. The process was completed by December 2012. The data collection process at baseline and end-line included quality checks and corrections. The baseline and end-line data were collected during the same months in 2011 and 2012, to adjust for seasonal variation in FV availability. Female heads of the households and selected individuals of all participating households signed written informed consent before data were collected. Data collection activities took place at the homes of the participants or as per their convenience.

#### Monitoring

Process documentation tools were used to monitor all key activities. For the community awareness sessions, the attendance was recorded. In addition, quality checks were put in place to ensure that reported activities were carried out.

### Roles and responsibilities of team members

#### Community volunteers

Community volunteers were recruited to carry out activities related to data collection and intervention delivery for the study period. All community volunteers were members of one of two community-based organizations: *Kerala Mahila Samakhya Society* (Kerala chapter of the ‘Education for women’s equality’ programme launched by the Government of India) or the *Kudumbasree mission*. Fifteen teams of two members, one each from the above organizations were finalized after completion of the training. Three teams were primarily responsible for administering the household staging tool. In order to minimize social desirability bias, they were allotted to administer the tool in areas other than their own. The remaining 12 teams participated in the general data collection. All data were checked for completion and correctness by the study coordinator and the principal investigator (PI; first author, MD). All incomplete data or incorrect or doubtful data fields were rechecked with the concerned household, either by the data collectors or the study coordinator during a subsequent visit or through phone contact. All community volunteers were also involved in the organization of the community awareness sessions in their respective areas. Further information about the community volunteers, their training and other details have been published elsewhere [24].

#### Counsellors

Doctoral students in Psychology with 3–6 months of practical experience in counselling were recruited as counsellors. They were not part of the community and were responsible for the counselling and community awareness sessions. They were responsible for conducting the face-to-face and telephonic counselling sessions, and the general awareness sessions for adults and children.

#### Study coordinator

The study coordinator, also not a community member, was employed to coordinate and oversee all project activities, and reported to the PI. He was responsible for conducting process and quality checks, and assisted in organizing the training sessions and data check points.

#### Training

Eight separate training sessions were organized for data collection tools and methods and intervention delivery and conducted by the PI and the study coordinator. Three of the data collection sessions were full-day of eight hours, while the remaining were half-day sessions lasting 4–5 hours.

Training sessions for intervention delivery were carried out separately and topped up with a refresher session after completion of baseline data collection. The community volunteers delivered all the intervention strategies, excluding conselling and awareness sessions which were delivered by the counselors. All study personnel spoke the local language, Malayalam.

In addition to the joint sessions with the community volunteers, the counsellors were trained in stages-of-change, its identification and relevance. Due to their educational background and experience, this was not a new concept to them. The teams were also given the information booklet to help them address any questions posed by household members. The counsellors were also trained separately on how to arrange interactive awareness sessions for the adults and children using flip charts and card games respectively. The study coordinator participated in all training sessions together with MD.

### Data analyses

Outcome measures and behaviour change variables are defined in Table 3 (Parts A & B) and other definitions relevant for the study are described in S1 Table. Intention-to-treat analysis was used to evaluate the effectiveness of the intervention at both household and individual level, with adjustments for cluster design effects. The intra-cluster correlation coefficient (ICC) was calculated for the *ayalkootam* level; and design effect was calculated using the formula: Design effect = 1 + (*m*– 1)*ICC, where *m* was the average cluster size. For analyses, the sample was weighted to make it representative for the households at the *grama panchayat* level. All dropouts were included in the analysis in the ‘no behaviour change’ category to avoid exaggerating the effect size. The unit of analysis was the household. Dietary intake measurement was only carried out for one adult individual per household and used as a measurement of household dietary intake by proxy.

**Table 3:** Definitions of outcome measures & behaviour change variables.

Variables	Definitions	Data collection/extraction
**A. Outcome measures** (as estimated procurement / consumption at baseline and end-line)		
*Fruit and vegetable intake* in servings (primary outcome):	Intake estimated in servings separately for fruits and vegetables for an individual adult member of the HH and considered as proxy for per capita FV intake in the recruited HH.	FV intake in servings was extracted separately for fruits and vegetables from two 24-hour recalls of all foods consumed by the selected healthy adult member between 25–45 years in each recruited household → the average of the two was estimated as daily fruit or vegetable intake for the selected individual
*2-week per capita fruit and vegetable procurement* in kilograms (secondary outcome)	Per capita estimate in kilograms of all FV procured or purchased for 2 weeks in each recruited HH.	Per capita calculations based on the 2-week FV procurement diary which enabled free listing for all FV procured or purchased for 2 weeks in each recruited household
*Monthly per capita salt*, *sugar and oil consumption in grams* (secondary outcome)	Monthly per capita estimate in grams for salt, free sugars and oils or fats consumed in each recruited HH.	Calculated from monthly consumption details reported for salt, free sugars and oils or fats by each recruited household in the general questionnaire
**B. Constructed behaviour change variables:** proportion of households who demonstrate behaviour change as defined in relation to each outcome measure		
*Vegetable intake*	Maintenance or increase to at least three vegetable servings by end-line	Constructed from the primary outcome variable: FV intake in servings
*Any fruit intake*	Consumption of any fruit (measured in serving) by end-line	Constructed from the primary outcome variable: *FV intake in servings*
*FV procurement*	Increase of at least 50% in the 2-week per capita HH procurement of fruits **or** vegetables.	Constructed from the secondary outcome variable: *2-week per capita fruit and vegetable procurement*
*SSO consumption*	Reduction of at least 10% in the monthly per capita HH consumption of salt, sugar **and** oil.	Constructed from the secondary outcome variable: *Monthly per capita salt*, *sugar and oil consumption in grams*

The per cent change from baseline (%Change) was estimated as the difference between end-line and baseline values as a percentage of the baseline value [(end-line–baseline/ baseline) x100]. Hence, even though the mean values at end-line may be lower than mean values at baseline, the overall %Change for the whole arm may be positive (or vice versa); since it is the mean of the %Change for individual households; as is the case for fruit and vegetable procurement (Results secction). Actual effect sizes described in the results were calculated as the sum of the effect size for %Change in intervention and control arm. Significant per cent change (%Change) from baseline in outcome variables was identified at p<0.05 by the null value being outside the 95% confidence intervals. All p-values comparing continuous variables between the two arms (difference of the difference) were based on the coefficient of linear regression for weighted samples adjusted for cluster design effect; and all proportions were compared using the chi-square. Statistical analyses were carried out using STATA data analysis software, version 12.1, owned by StataCorp, Texas, USA and licensed to Karolinska Institutet, Stockholm, Sweden.

### Deviations from protocol

There were two deviations from protocol.

1. Intervention components: One telephonic counselling and two telephonic reminders by the community volunteers were scheduled over the one-year period. It was often difficult to contact the participants over phone. The reasons cited were not being available at home; shared mobile phones where another family member had the mobile; or change of mobile numbers, which was a frequent occurrence. For those households who could not be contacted over phone, community volunteers or the study coordinator conducted reminder home visits.
2. Data analysis: Fruit intake was initially defined as part of FV intake as maintenance or increase to at least two fruit servings by end-line. Due to very low baseline and end-line fruit intake the numbers were inadequate for one or two daily fruit intake servings. Hence the outcome variable was split into *vegetable intake* and *any fruit intake* instead (Table 3, Part B).

### Ethical consideration and trial registration

All data collections and study procedures were conducted according to the guidelines laid down by the Indian Council of Medical Research. The Institutional Ethics Committee of Sree Chitra Tirunal Institute for Medical Sciences and Technology, Trivandrum approved all the procedures involving the study participants related to the conduct of the cRCT (SCT/IEC-357/MAY-2011; Date: 11/06/2011). Participants were given information sheets detailing the nature of the study and their role; the voluntary nature of their participation; and their right to withdraw from the study at any point. Participants were also given an opportunity to ask questions and seek clarifications, before recruitment. Both the female heads of the households and the selected individual in each household signed the written informed consent. The awareness sessions were open to all adults and children (card games) in the community irrespective of their participation in the trial and were conducted under the auspices of the *ayalkootams*. No separate written consent was signed for participation in the awareness sessions. The study was registered prospectively under the Clinical Trial Registry of India (CTRI/2011/06/001839; Date: 28/06/2011) [38].

## Results

### Participant flow

**Fig 2:**
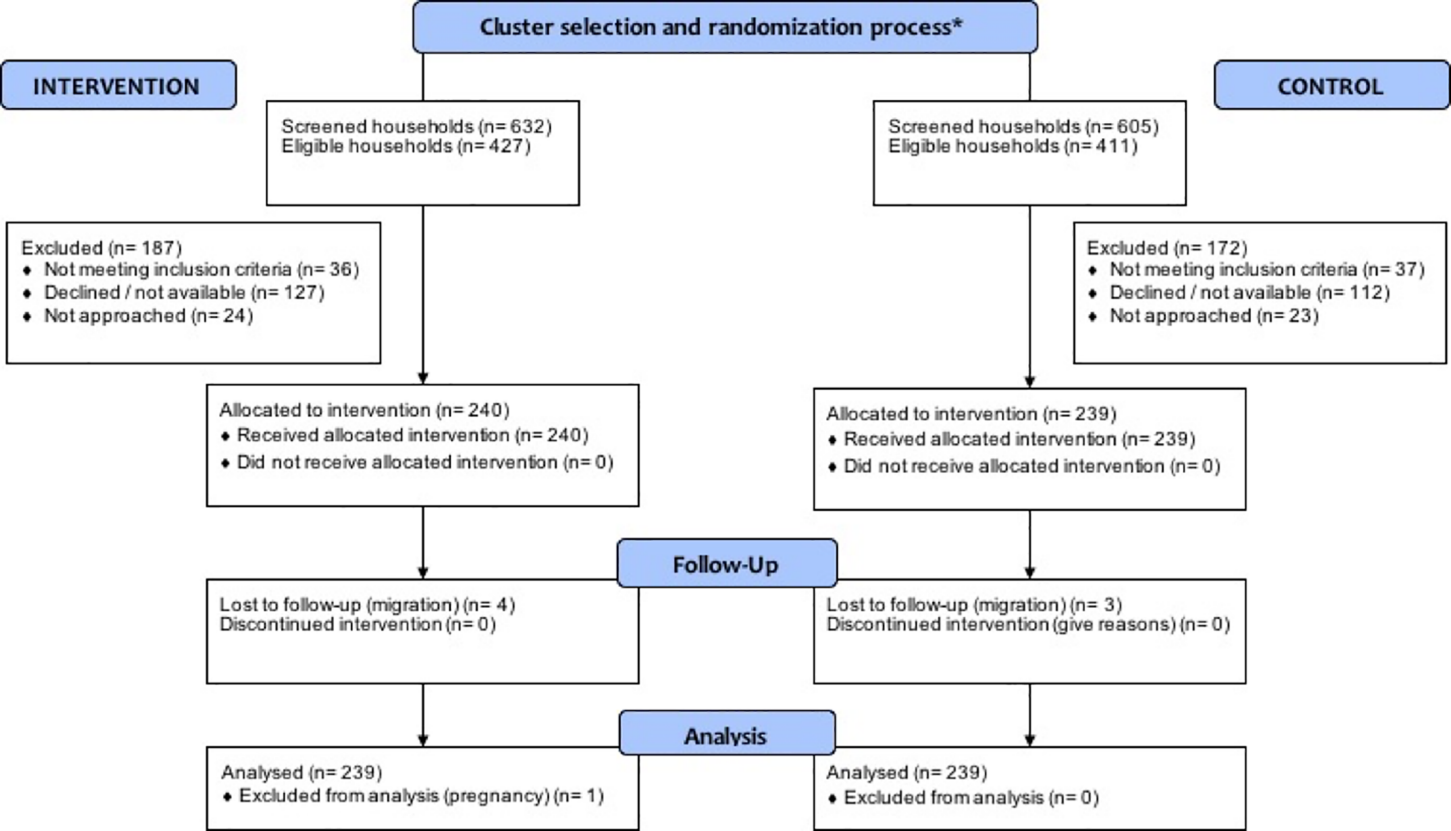
Flow diagram showing participant flow through the trial.

Participant flow through the trial is described in a flow diagram as recommended in the CONSORT guidelines 2010 (Fig 2)

### Recruitment and participation

In total 1237 households were screened, and 838 households were found to be eligible. Of these, 791 households were approached and 479 recruited for the baseline survey (Fig 2). We stopped approaching the households once we recruited the required number. The response rates for males (about 40%) were much lower compared to females (at least 90%) in both intervention and control arms. A total of 471 households from both intervention and control arms completed the end-line survey after one year. There were four and three dropouts (due to change of address or employment), respectively, in the intervention and control arms; and one exclusion (pregnancy) in the intervention arm. For the intention-to-treat analysis, all baseline households, barring the exclusion (n = 478), were included.

**Fig 3:**
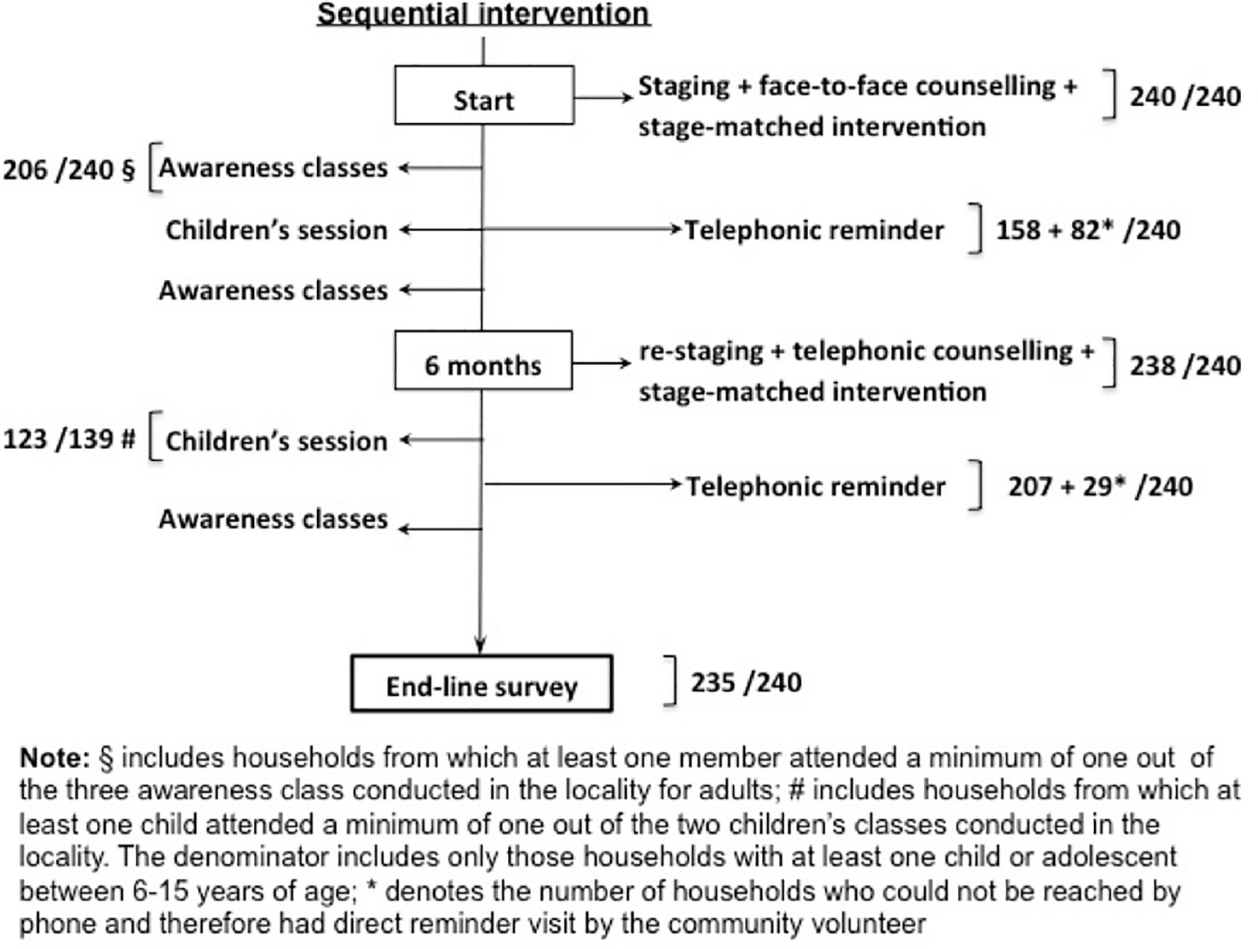
Participation in various stages of the intervention in the intervention arm.

Participation in intervention components are shown in Fig 3. Overall, participation was high, except for telephone reminders. Participation in the awareness and children’s sessions were 85% and 88% respectively. The telephone reminders had to be complemented with reminder visits in those who could not be reached by phone.

### Baseline data

#### Demographic characteristics

Overall, 48% and 52% of households were in the lower and higher middle SES, respectively. The mean household size was 4.6. The demographic characteristics were comparable across the intervention and control arms (Table 4). The baseline diet-related characteristics were also comparable and are described in S2 Table.

**Table 4:** Distribution of demographic characteristics in two arms at baseline.

Characteristic	Intervention n = 239 (%)	Control n = 239 (%)
*1*. *Age group (in years)* †		
25–35	115 (48%)	121 (51%)
36–45	124 (52%)	118 (49%)
*2*. *Gender* †		
Male	98 (41%)	96 (40%)
Female	141 (59%)	143 (60%)
*3*. *Marital status* †		
Unmarried	26 (11%)	24 (10%)
Married	203 (85%)	207 (87%)
Divorced, separated or widowed	10 (4%)	8 (3%)
*5*. *Highest educational qualification in the household*		
≤ 10 years of formal education	97 (41%)	82 (34%)
> 10 years of formal education	142 (59%)	157 (66%)
*7*. *Occupation of the main income earner of the household*		
No paid employment	34 (14%)	26 (11%)
Paid employment (irregular)	161 (66%)	147 (61%)
Paid employment (regular)	44 (18%)	66 (28%)
*8*. *Household size*		
≤ 4 members	150 (63%)	131 (55%)
> 4 members	89 (37%)	108 (45%)
*9*. *Monthly per capita household expenditure* ‡		
Low-middle expenditure group	128 (54%)	111 (46%)
High-middle expenditure group	111 (46%)	128 (54%)
*10*. *Household NCD status* §		
No NCDs in the household members	112 (47%)	133 (56%)
At least one HH member has NCD	127 (53%)	106 (44%)
*11*. *Children aged 11 years or less in the household*		
No	114 (48%)	101 (42%)
Yes	125 (52%)	138 (58%)
*12*. *Adolescents aged 12–18 years in the household*		
No	142 (59%)	148 (62%)
Yes	97 (41%)	91 (38%)

† Variables related to selected individual in the household.

‡ Monthly per capita household expenditure was the proxy for socio-economic status. High- and low- middle expenditure groups were divided by the median value (INR. 1417).

§ NCDs considered for the variable are diabetes, hypertension, hypercholesterolemia on medication, cancer or heart disease.

Abbreviations: NCD: non-communicable diseases.

#### Food-related characteristics

SSO was mainly added to food during the cooking process. Adding salt to rice was a fairly common practice (30%) and only 19% of the individual participants added salt to their rice before eating. Eighty-eight percent reported seasoning their dishes with oil and other condiments while cooking. Only ten individuals habitually consumed a non-home-cooked lunch, usually at or close to their workplace. Most of the employed men (in the study) carried their lunch packets from home.

#### Distribution of stages-of-change

The distribution of stages-of-change in the intervention arm across the three time-points: baseline; 6-months; and end-line; and the status of change across these time points in terms of progress, relapse or no change as measured by the household staging tool are shown in S1 Fig. Overall, households in action stage increased from six per cent (15/240) at baseline to 87% (208/240) by end-line. There were both progress and relapse across the three time-points, with the majority of the households in intention stage at baseline (138/240 = 58%); and in action stage at six-months (135/240 = 56%) and end-line (208/240 = 87%).

### Outcomes and estimations: Results of the intention to treat analysis

Table 5 describes the primary and secondary outcome measures with baseline and end-line values, along with per cent change from baseline with p-value showing difference between intervention and control arms. Monthly per capita salt, sugar and oil consumption were significantly lower in the intervention arm at end-line, while the control arm demonstrated an increased consumption for all three.

**Table 5:** Mean (SEM) for baseline, end-line & percent change in outcome variables: Results of the intention-to-treat analysis.

Outcome variable	Intervention (n = 239)			Control (n = 239)			
	Baseline	End-line	%Change‡	Baseline	End-line	%Change‡	p-value § (%Change)
**Daily fruits & vegetables intake**							
Fruit intake in servings	0.21	0.30	+12.46*	0.22	0.24	+6.62*	0.137
	(0.04)	(0.04)	(3.13)	(0.02)	(0.03)	(2.27)	
Vegetable intake in servings	1.83	1.86	+13.99*	1.76	1.80	+13.66*	0.967
	(0.10)	(0.11)	(5.45)	(0.12)	(0.11)	(6.02)	
**Two-week per capita fruit and vegetable procurement**							
Fruit procurement in kg	1.58	1.56	+12.27*†	1.17	1.08	+5.30	0.324
	(0.11)	(0.12)	(5.36)	(0.08)	(0.08)	(4.49)	
Vegetable procurement kg	1.30	1.24	+10.19†	1.06	0.66	-8.97*	0.008
	(0.09)	(0.09)	(5.24)	(0.08)	(0.07)	(4.33)	
**Monthly per capita salt, sugar & oil consumption**							
Salt consumption in grams	303	167	-34.37*	246	252	+11.15	<0.001
	(16)	(10)	(5.39)	(8)	(11)	(5.66)	
Sugar consumption in grams	727	494	-17.95*	618	653	+21.94	<0.001
	(37)	(24)	(3.89)	(30)	(40)	(7.80)	
Oil consumption in grams	464	356	-11.98*	440	519	+35.51*	<0.001
	(22)	(21)	(3.19)	(20)	(20)	(7.29)	

‡ The per cent change from baseline (% Change) was estimated as the difference between end-line and baseline values as a percentage of the baseline value [(end-line–baseline/ baseline) x100].

† Even though the mean values at end-line may be lower than mean values at baseline, the overall % Change for the whole arm may be positive (or vice versa); since it is the mean of the %Change for individual households; as is the case for fruit and vegetable procurement.

§ p-values for difference of the difference (i.e. difference in %change between the intervention and control arms) were based on the coefficient of linear regression for weighted samples adjusted for cluster design effect.

* p<0.05.

Abbreviation: SEM–Standard error of mean.

Daily fruit intake showed significant increase in terms of per cent change from baseline in both the intervention and control arms (12.5% and 6.6%, respectively), with no significant difference between the two arms. Vegetable intake showed an overall significant increase in both arms (14%) from baseline. The two-week per capita fruit procurement also showed an increase from baseline in both arms (12.3% and 5.3%, respectively), which was significant only for the intervention arm, but the difference between the two arms was not significant. The two-week per capita vegetable procurement showed an increase in the intervention arm (not significant) in terms of percentage change from baseline and a significant decline in the control arm (10% and *minus* 9%, respectively), making the difference between the two arms significant.

Per capita monthly salt, sugar and oil consumption showed a significant decrease in the intervention arm from baseline to end-line, and a significant increase in oil consumption from baseline in the control arm. The difference between the two arms was also significant. Considering the temporal trend in the control arm, the actual effect sizes were as follows: vegetable procurement: *an overall increase by* 19%; consumption of salt: *an overall decrease by* 45%; sugar: *an overall decrease by* 40%; and oil: *an overall decrease by* 48% at the household level. The mean coconut consumption was also significantly reduced from 1.4 to 1.0 coconut per day in the intervention households compared to no change in the control households (not in table).

The proportions of households demonstrating behaviour change is described Table 6. Behaviour change for FV intake and procurement and SSO consumption are defined in Table 3 (Part B).

**Table 6:** Proportion of households showing change in behavioral outcomes.

Behavioral outcome	Intervention n = 239	Control n = 239	p-value*
**Fruits & vegetables intake & procurement (as defined by protocol)** †			
*1*. *Any fruit intake by end-line*			
No	168 (70%)	181 (76%)	
Yes	71 (30%)	58 (24%)	0.249
*2*. *Maintained or increased to at least three daily vegetable servings by end-line*			
No	197 (82%)	196 (82%)	
Yes	42 (18%)	43 (18%)	0.726
*3*. *Increased 2-week per capita fruit procurement by at least 20%*			
No	159 (67%)	164 (69%)	
Yes	80 (33%)	75 (31%)	0.807
*4*. *Increased 2-week per capita vegetable procurement by at least 20%*			
No	158 (66%)	199 (83%)	
Yes	81 (34%)	40 (17%)	0.017
**Salt, sugar & oil consumption (as defined by protocol)** †			
*5*. *Reduced monthly per capita salt consumption by at least 10%*			
No	51 (21%)	167 (70%)	
Yes	188 (79%)	72 (30%)	<0.001
*6*. *Reduced monthly per capita sugar consumption by at least 10%*			
No	92 (38%)	152 (64%)	
Yes	147 (62%)	87 (36%)	0.0002
*7*. *Reduced monthly per capita oil consumption by at least 10%*			
No	113 (47%)	171 (72%)	
Yes	126 (53%)	68 (28%)	<0.001
**Combined behavior change variables** †			
*8*. *FV procurement*: *increased 2-week per capita fruit* *or* *vegetable procurement by at least 50%*			
No	158 (66%)	197 (82%)	
Yes	81 (34%)	42 (18%)	0.026
*9*. *SSO consumption*: *reduced monthly per capita salt*, *sugar* *and* *oil consumption by at least 10%*			
No	155 (65%)	219 (92%)	
Yes	84 (35%)	20 (8%)	<0.001

* All p-values are based on weighted samples using chi-square test taking into account cluster design.

† All variables are defined in Table 3 (Part B).

Abbreviations: FV–fruit and vegetable; SSO–salt, sugar and oil.

### Harms

No specific harms or unintended adverse effects were identified as a consequence of the trial. Diet and dietary behaviours have a cultural and social meaning beyond that of just consuming ‘food’. Any harms from the intervention mainly amounted to discomfort of perceived invasion of privacy while answering questions related to individual or household dietary practices and behaviours. This is particularly relevant when coupled with lack of resources in households or the unintended labelling of certain dietary behaviours as healthy or unhealthy. Every attempt was made to ensure privacy and confidentiality during data collection and intervention delivery.

## Discussion

### Key findings

The intention-to-treat analysis showed a modest increase in fruit and vegetable intake from baseline in both intervention and control arms, but no significant difference between the two arms. However, there was an increase in vegetable procurement in the intervention arm compared to the control arm with 34% of all households in the intervention arm showing an increase in FV procurement. Salt, sugar and oil consumption demonstrated the greatest reductions with actual effect sizes exceeding 40%.

### Interpretation of key findings

#### FV intake and procurement

FV intake was very low at baseline. Only one per cent of the study population consumed five daily servings of fruits or vegetables. There is a wide variation in reported FV intake for Kerala from different studies, ranging from 13% to 53% [16, 34, 39]. Our findings were somewhat comparable to baseline levels in only one study conducted near Delhi, which reported six per cent consumption of five FV servings among men and three per cent among women [19].

Intervention studies on FV intake among adults have been conducted in diverse settings: through primary care services in the UK [40, 41]; in the workplace in South Eastern Brazil [42]; and in communities in Japan, Panama and Trinidad and Tobago [43–45]. White *et al* used goal setting, behaviour planning and peer support, group discussions and weekly self-tracking for six-weeks; followed by extension of behaviour change to other family members and weekly meetings through participant initiative for the subsequent six-months. They were able to demonstrate an increase in FV intake at six weeks, but this was not sustained over the next six months [45]. Takahashi *et al*. used two 15-minute individual dietary counseling sessions, one group lecture, two newsletters; explicit sub-goals and leaflets with nutrition and cooking hints and computer tailored dietary information. They were able to demonstrate a sustained improvement in FV intake supported by biomarkers (carotene and vitamin C) over four years [43, 44].

However, there is a lack of similar studies in the Indian context. Most studies looked at change in NCD risk factors or prevalence of diabetes or hypertension and have not measured or reported FV intake [18, 46]. A community-based study near Delhi was able to demonstrate only a modest increase of three per cent in men and five per cent in women for FV intake, in spite of using a multi-component community approach [19].

Our study results showed a disconnect with an increase in FV procurement and lack of significant change in FV intake. Two crucial steps are necessary to increase FV intake. The first step is an increase in purchase or procurement, often involving higher monetary costs. Healthier food options including fruits and vegetables are more expensive in most settings [47, 48], unless they are made available under special schemes or subsidies [49]. Our intervention was able to demonstrate a significant increase in FV procurement. This was also reflected in the composite FV procurement variable, which showed a significantly higher proportion of intervention households demonstrating an increase in either fruit or vegetable procurement by at least 50% (Table 6). The decrease in the control arm was probably a reaction to the price rise, which the intervention households were able to withstand and even increase from baseline levels (see section below on secular trends). Use of locally available FV, which was one of our intervention strategy, was possibly a behaviour shift mechanism employed by households to tide over the increased FV prices. This behaviour has to be encouraged through interventions or other policy-level efforts. It has the added advantage of being relatively unaffected by price rise and of being pesticide-free, which was a serious concern raised by households during our formative interviews.

The second step that must follow is that the significant increase in procurement should translate to a significant increase in consumption, which we were unable to conclusively demonstrate in this study. We’ve identified two possible explanations. 1) The pre-trial phase of our study revealed low risk perception, low outcome expectations and low self-efficacy regarding the need and ability to make and sustain life-style changes [15]. Hence, translating the increased FV procurement to increased FV consumption in individuals is likely to take more time than the 1-year period that was available for the trial. 2) The female head of the household responsible for procurement in the household and the individual for whom intake was measured need not be the same; as the latter was selected randomly using Kish methodology.

#### SSO consumption

Our intervention was able to significantly reduce SSO consumption in the intervention arm, while the temporal trend in the control arm was an increase in consumption over the same time period. Households were able to use the kit very effectively to make slow and sustainable changes in the use of SSO during food preparation. The components of the household kit were tailored to individual household needs based on their size. The kit was also designed using locally available containers and measures in such a way, that it could be easily incorporated into the regular food preparation activities of a household.

SSO was mainly added to food during the cooking process; and each individual had very little to do with it. Moreover, most of the employed men (in the study) carried their lunch packets from home. In this scenario, it made sense to tackle SSO consumption through the household food preparation process. A community-based dietary intervention study in Japan also showed dramatic and sustained results in sodium consumption for over four years by targeting the consumption of salted pickles, fish and other items [43, 44].

The low risk perception that correlates with the low priority given to health in the food decision-making process is an important factor to be considered. Health became an issue only when it was seriously compromised; or was perceived to be at risk of being seriously compromised; as demonstrated by the NCD status of household members, which was a significant predictor for reduction of SSO consumption. About half the households in the cRCT had at least one member who had a diagnosed NCD. This meant that these households were exposed to the standard advice from medical practitioners about reducing salt, sugar and oil, with respect to hypertension, diabetes and hypercholesterolemia respectively. So, households were already sensitized to the need to reduce SSO consumption, but not to increase FV intake as there is no such standard practice regarding FV. Hence, the intervention should have focused more on household strategies to increase FV intake, particularly in the absence of such prior sensitization.

#### Secular trends during the intervention period

There were two important events during the intervention period. The National Program for prevention and control of Cancer, Diabetes, Cardiovascular diseases and Stroke (NPCDCS) was launched and implemented across Kerala, including *Thiruvananthapuram* district [50]. In 2008, the pilot phase of the NPCDCS was launched in seven states, initially in one district each, including *Thiruvananthapuram* district. Awareness sessions aired in the state-run radio and television channels addressed all NCD risk factors including diet (both FV and SSO), together with other aspects of a healthy diet. Together with the National Rural Health Mission, the programme had significantly stepped up mass media campaigns, over the last two years. The campaigns included for example, newspaper articles, radio advertisements, radio talk shows and billboard campaigns; and could be accessed by both intervention and control arms equally.

There had also been a significant increase in prices of commonly used fruits and vegetables, ranging from 25–100% in many cases, over the intervention period from July 2011 to September 2012. Kerala state also faced another unique problem as domestic production of vegetables is very low, and therefore almost all vegetables have to be imported from neighbouring states. Fruits were generally available locally, but were seasonal. As a response to the price rise and the strong public sentiment it had generated, some *panchayats*, including two intervention and one control *panchayat* had started an innovative community farming initiative around August-September 2012. This coincided with the point of time when our end-line survey was conducted.

### Limitations

#### Dietary intake and procurement measurement

Many studies use the generic or modified version of the STEPS instrument developed by WHO, which included four items to assess FV intake. We used two detailed 24-hour recalls, one on a weekday and the other on a weekend; and incorporated the multiple-pass method to improve accuracy [51]. Short FV screeners to measure FV intake have been found to systematically overestimate, underestimate or measure similar values when compared to repeated 24-hour recalls or diet records; based on the items included and the wording of the questions [52, 53]. However, all dietary intake assessment method without a biochemical component are prone to social desirability bias and some degree of under- or over-reporting.

The quantification of food procurement is usually done through supermarket receipts or estimated from expenditure [51]. In our setting, such quantification was not feasible as most local markets and small stores did not provide receipts. Moreover, we wanted to capture both purchase from market and use of local FV. Hence, we developed a 2-week procurement diary, where the female head of the household could enter all the purchases or procurements made per day in terms of its weight; or in terms of price or amount in numbers if the weight was not known. The investigator estimated the 2-week procurement based on local prices at the time of the survey and average weight of medium sized items.

#### Male participation

Low male participation was a problem, and this has been the experience in other studies as well [54, 55]. Participation response rates for men were only 40% in both arms. However, participation of men was essential to ensure that the study was acceptable in households. Husband’s preferences were found to be a key consideration in the dietary decision-making process; and we took additional effort to ensure that men in the selected households were able to participate in as many of the intervention strategies as possible, particularly community awareness and counselling sessions. We also ran the risk of the whole project being labelled as a ‘women’s issue’ with very little or negligible male participation if all the study personnel were women. As the lay community volunteers were all women we decided to recruit men for the positions of counselors and study coordinator, to encourage more male participation within the households and communities.

#### ‘Real life’ intervention trial

Conducting a ‘real life’ cRCT had its own challenges at different levels. Here is a brief description of some incidents over the one-year period, in order to understand the complexity of conducting such a study. Initially, we talked to the *panchayat* leaders and administrators, including the elected representatives of the different wards and the administrators of the *Kudumbasree* and the different *ayalkootams*. The representatives of the chosen wards or *ayalkootams* often used the intervention to gain more attention from the public, while the others were not happy about being excluded. Similarly, in some *ayalkootams*, we had to explain that even though only a few households received the household kit after each survey; all would receive the same by end-line. Moreover, we also had to make provisions for methodological challenges that arose during the intervention. The use of telephone reminders did not work as expected and had to be supplemented with home visits. Challenges related to the use of mobile phones in interventions, arising from frequent change of numbers or shared usage are not new and have been reported in other studies [15, 56].

### Generalizability

We did not include households with low and high SES. Even though middle class in Kerala comprises 85% of the population [32], this would still affect the generalizability of the results. We did however, conduct a multi-stage stratified random sampling process and weighted analysis to improve generalizability.

### Strengths of the study

The major strength of the study is the understanding of the context gained through formative research, which subsequently informed the design and conduct of the cRCT. The cRCT itself was designed with a lot of attention to the randomization and selection processes to minimize bias. The study design also had to incorporate elements, which would make long-term sustainability possible. To this end, the study made maximal use of existing infrastructures in the form of community volunteers, self-help group networks and existing administrative divisions. The use of community volunteers ensured better acceptability and minimized both non-response as well as dropout rates as they were living in these communities and knew most of the study participants. Moreover, the household kit was designed based on the response from focus group discussions and in-depth interviews conducted as part of formative research.

We also utilized the potential of the communities, peers and families, who have often been used as ‘change agents’ for many community-based interventions [45]. In complex multi-cultural societies like India and Kerala, the approval or support of these ‘change agents’ is often crucial. It was not enough to increase awareness and educate the target households alone, we also had to devise strategies to enable communities to be more receptive, so that these changes became possible and even expected. Hence, the communities and other household members, particularly children, were also included as part of the broader intervention package. Due to the high prevalence of NCDs [15, 16, 57], most people were partially aware of this reality, particularly the relation between SSO and NCDs, and were concerned for their children, which we identified as a powerful motivator.

### Implications of the findings

The very low baseline FV intake calls for serious health promotion activities to improve awareness; and to increase demand and stabilize supply of FV in communities. The community farming initiatives that were started by many *panchayats* in August-September 2012 is a step in the right direction. Moreover, lack of any pro-active government policies or interventions could lead to an inequitable FV intake differentiated by socio-economic strata and gender.

The household kit and other household-centered strategies, like use of local FV, can be implemented at the *panchayat* level to increase fruit intake and FV procurement, and to reduce SSO consumption. Specific strategies should be evolved to engage both men and children actively in the household behaviour change process. Ways to address individual FV intake behaviour through the household should also be explored further. Low SES households need specific policy measures, particularly targeted food subsidies, if they are to have a comparable chance of success as other SES groups.

It is important, both in intervention studies and mass media campaigns, to address the lack of or limited awareness of the link between diet and NCD, particularly related to FV; the risk of NCDs for the family members; and the benefits of simple prevention measures. Introduction of local markets in difficult geographic locations, or improving cost-effective transportation means, either for people to access markets or for foodstuffs to be brought to the people, could change behavioural outcomes within very short time periods.

The use of existing community networks and community volunteers, represent a tremendous linkage opportunity for a country like India struggling with unemployment on the one hand and lack of adequate health manpower on the other. This study has demonstrated that community volunteers particularly in a highly literate state like Kerala, can deliver complex interventions; provided they receive adequate training and the knowledge has been appropriately translated into a form that can be used by them. This would also make such interventions potentially sustainable in the long run as training of community volunteers and constant reinforcement of this knowledge would raise the basic knowledge and practice levels in communities of which they are a part. However, challenges associated with maintaining volunteer motivation [58] and finding a sustainable remuneration model [59] for their continued work are well known and are applicable in this setting as well. This study mainly looked at the feasibility of delivering complex interventions through exisiting networks and community volunteers and focused on the choice of strategies and delivery modalities in terms of their potential long-term sustainability. However, the sustainability aspect needs to be evaluated carefully before a scale-up of this intervention can be considered.

## Conclusions

This work represents a significant contribution to the existing body of knowledge relating to the understanding and promoted change of dietary behaviour relating to FV intake and procurement and SSO consumption using context-specific household-centred intervention strategies. The strategies and developed tools enabled a large reduction in SSO consumption of 40–48% and improvement in FV procurement in the intervention arm over and above the temporal trend as manifested in the control arm. However, we were not able to demonstrate significant changes in FV intake in the intervention arm compared with the control arm, in spite of very low baseline levels. The difference in the behavioural processes at the household versus individual level which govern SSO consumption and FV intake behaviours possibly account for this disparity. Our major assumption that dietary behaviours in this setting had a strong household component was justified by the results of the cRCT. However, we need to explore FV intake behaviour further to identify strategies or components that could make a greater difference. As one of the first studies in this field within this setting, we have learnt important lessons that we can take forward, while at the same time improving our understanding of human dietary behaviour and how that can be changed to improve health.

## Supporting information

S1 Checklist CONSORT 2010 checklist: Changing household dietary behaviours in rural Kerala, India. (PDF)

S1 Table Relevant definitions for the study. (PDF)

S2 Table Distribution of diet-related characteristics in two arms at baseline. (PDF)

S1 Fig Sequential stage-matching of interventions in the intervention arm. (TIFF)

## Abbreviations

NCD: Non-communicable diseases
HH: Household
FV: Fruit & vegetable
SSO: salt, sugar & oil
cRCT: Cluster randomized controlled trial
