# Supplementary material for: Changing household dietary behaviours through community-based networks: A pragmatic cluster randomized controlled trial in rural Kerala, India
Source: PLoS One. 2018 Aug 22;13(8):e0201877. doi: 10.1371/journal.pone.0201877 (PMC6104953; doi:10.1371/journal.pone.0201877)
Supplement: S1 Checklist — (PDF) [file pone.0201877.s001.pdf]

CONSORT 2010 checklist: Changing household dietary behaviours in rural Kerala, India.

Section/Topic	Item No	Checklist item	Reported in: Section [page no.]
**Title and abstract**			
	1a	Identification as a randomised trial in the title: Pragmatic cluster randomized controlled trial	Title [1]
	1b	Structured summary of trial design, methods, results, and conclusions (for specific guidance see CONSORT for abstracts)	Abstract [2-3]
**Introduction**			
Background and objectives	2a	Scientific background and explanation of rationale	Background & rationale [4-6]
	2b	Specific objectives or hypotheses	Objectives [7]
**Methods**			
Trial design	3a	Description of trial design (such as parallel, factorial) including allocation ratio	Study plan & sampling procedure [8-10]
	3b	Important changes to methods after trial commencement (such as intervention components), with reasons	Deviation from protocol [21-22]
Participants	4a	Eligibility criteria for participants	Study plan & sampling procedure [9-10]
	4b	Settings and locations where the data were collected	Study setting [7]
Interventions	5	The interventions for each group with sufficient details to allow replication, including how and when they were actually administered	Description of intervention [12-16]
Outcomes	6a	Completely defined pre-specified primary and secondary outcome measures, including how and when they were assessed	Data analysis [19-21]
	6b	Any changes to trial outcomes after the trial commenced, with reasons	Deviation from protocol [21-22]
Sample size	7a	How sample size was determined	Sample size [10-11]
	7b	When applicable, explanation of any interim analyses and stopping guidelines	--
**Randomisation**			
Sequence generation	8a	Method used to generate the random allocation sequence	Study plan & sampling procedure [8]

	8b	Type of randomisation; details of any restriction (such as blocking and block size)	Study plan & sampling procedure [9]
Allocation concealment mechanism	9	Mechanism used to implement the random allocation sequence	Study plan & sampling procedure [9]
Implementation	10	Who generated the random allocation sequence, who enrolled participants, and who assigned participants to interventions	Study plan & sampling procedure [8]
Blinding	11a	If done, who was blinded after assignment to interventions (for example, participants, care providers, those assessing outcomes) and how	--
	11b	If relevant, description of the similarity of interventions	--
Statistical methods	12a	Statistical methods used to compare groups for primary and secondary outcomes	Data analysis [20]
	12b	Methods for additional analyses, such as adjusted analyses	Data analysis [20]
**Results**			
Participant flow (a diagram is strongly recommended)	13a	For each group, the numbers of participants who were randomly assigned, received intended treatment, and were analysed for the primary outcome	Participant flow [23-24]
	13b	For each group, losses and exclusions after randomisation, together with reasons	Participant flow [23-24] & Recruitment & participation [24-25]
Recruitment	14a	Dates defining the periods of recruitment and follow-up	Data collection – Process [17]
	14b	Why the trial ended or was stopped	Data collection – Process [17]
Baseline data	15	A table showing baseline demographic and clinical characteristics for each group	Baseline data [26-28]
Numbers analysed	16	For each group, number of participants (denominator) included in each analysis and whether the analysis was by original assigned groups	Participant flow [23-24] & Recruitment & participation [24-25]
Outcomes and estimation	17a	For each primary and secondary outcome, results for each group, and the estimated effect size and its precision (such as Standard Error of Mean)	Outcome estimation [28-29]
	17b	For binary outcomes, presentation of both absolute and relative effect sizes is recommended [Relative effect size]	Outcome estimation [29]
Ancillary analyses	18	Results of any other analyses performed, including subgroup analyses and adjusted analyses, distinguishing pre-specified from exploratory	--
Harms	19	All important harms or unintended effects in each group (for specific guidance see CONSORT for harms)	Harms [32]

**Discussion**			
Limitations	20	Trial limitations, addressing sources of potential bias, imprecision, and, if relevant, multiplicity of analyses	Limitations [37-39]
Generalisability	21	Generalisability (external validity, applicability) of the trial findings	Limitations – Generalizability [39]
Interpretation	22	Interpretation consistent with results, balancing benefits and harms, and considering other relevant evidence	Interpretation of key findings [32-36]
**Other information**			
Registration	23	Registration number and name of trial registry	Trial registration [1]
Protocol	24	Where the full trial protocol can be accessed, if available	Other information [43]
Funding	25	Sources of funding and other support (such as supply of drugs), role of funders	Other information [43]

Note: All modifications to the CONSORT checklist 2010 are marked in blue font
