# Supplementary material for: Changing household dietary behaviours through community-based networks: A pragmatic cluster randomized controlled trial in rural Kerala, India
Source: PLoS One. 2018 Aug 22;13(8):e0201877. doi: 10.1371/journal.pone.0201877 (PMC6104953; doi:10.1371/journal.pone.0201877)
Supplement: S1 Table — (PDF) [file pone.0201877.s002.pdf]

S1 Table: Relevant definitions for the study

Variables	Definitions	Data collection/extraction
Household	A household is defined as comprising all individuals who live under one roof and share a common kitchen	--
Fruits & vegetables	From a nutritional point of view, fruits and vegetables were defined as low energy-dense foods relatively rich in vitamins, minerals and other bioactive compounds and a good source of dietary fiber (Agudo 2005). For the purpose of this study vegetables were defined as "vegetables and foods used as vegetables" (Agudo 2005, p. 6). This includes fresh pulses, sprouts, and fruits used as vegetables, for example tomatoes, raw mangoes and papaya while, potatoes, tapioca (cassava) and dried pulses were excluded. The term, fruits referred to "the mature ovary of plant, which encloses the seeds" (Agudo 2005, p. 4). Only 100% fruit juice was considered as fruit (Agudo 2005). Foods derived from fruits, like jams, jellies and preserves were not included.	24-hour dietary recall & 2-week FV procurement
One serving of vegetables	A volume (250 ml) of raw leafy vegetables or half this volume (125 ml) of cooked or chopped vegetables accounted for approximately 80g; and was considered as one serving (Agudo 2005). For this study, investigators used two types of serving spoons usually used by the people in this setting to measure serving sizes. One deep serving spoon (*thavi*), which measured approximately 80g of cooked vegetables, was used to measure one serving for curry items. Three heaped tablespoons, also approximately measured 80g and was used to measure one serving for dry vegetable dishes.	24-hour dietary recall
One serving of fruits	For fruits, the edible part of one whole medium-sized fruit or two smaller sized fruits accounts for at least 80g and made one serving (Agudo 2005). A fruit chart describing one serving for all locally used fruits was used to assess fruit intake.	24-hour dietary recall
Free sugars:	The term ''free sugars'' referred to all monosaccharides and disaccharides added to foods by the manufacturer, cook or consumer, plus sugars naturally present in honey, syrups and fruit juices (WHO 2003). In the study context, this included refined sugar, jaggery, honey and jams. Sugar content of jams commonly range from 60-70%, and could be as high as 80%. For this study, we have estimated sugars in jams at 65%.	General Household Questionnaire
Fats and oils:	All oils and fats used for cooking were included. In the study context, this included clarified butter (ghee) and hydrogenated vegetable oils (*vanaspati*).	General Household Questionnaire

References: **Agudo A (2005)** Measuring intake of fruit and vegetables. Background paper for the Joint FAO/WHO Workshop on Fruits and Vegetables for Health, 1-3 September 2004, Kobe, Japan. World Health Organization, Geneva.; **World Health Organization (2003)**. Diet, nutrition and the prevention of chronic diseases: report of a joint WHO/FAO joint consultation. WHO Technical Report Series 916. Geneva: World Health Organization.
