# Supplementary material for: Changing household dietary behaviours through community-based networks: A pragmatic cluster randomized controlled trial in rural Kerala, India
Source: PLoS One. 2018 Aug 22;13(8):e0201877. doi: 10.1371/journal.pone.0201877 (PMC6104953; doi:10.1371/journal.pone.0201877)
Supplement: S2 Table — (PDF) [file pone.0201877.s003.pdf]

S2 Table: Distribution of diet-related characteristics in two arms at baseline

Characteristic	Intervention n = 239 (%)	Control n = 239 (%)	p-value*
1. Access to nearest local market			
Accessible by walk	183 (77%)	189 (79%)	
Not accessible by walk	56 (23%)	50 (21%)	0.618
2. Habit of consuming main meals †			
With family	186 (78%)	202 (85%)	
Alone or with others	53 (22%)	37 (15%)	0.276
3. Type of diet consumed †			
Complete vegetarian	8 (3%)	7 (3%)	
Partial- or non-vegetarian	231 (97%)	232 (97%)	0.782
4. Type of oil used for cooking			
Only coconut oil	216 (90%)	222 (93%)	
More than one type of oil	23 (10%)	17 (7%)	0.206
5. Daily fruit intake of at least 2 servings			
No	236 (99%)	238 (99.6%)	
Yes	3 (1%)	1(0.4%)	0.327
6. Daily vegetable intake of at least 3 servings			
No	201 (84%)	206 (86%)	
Yes	38 (16%)	33 (14%)	0.798
7. Use of locally available fruits			
No	180 (75%)	195 (82%)	
Yes	59 (25%)	44 (18%)	0.243
8. Use of locally available vegetables			
No	204 (85%)	214 (90%)	
Yes	35 (15%)	25 (10%)	0.229

* All p-values are based on weighted samples using chi-square test taking into account cluster design; † Variables related to selected individual in the household.
