# Supplementary figures and images for: Changing household dietary behaviours through community-based networks: A pragmatic cluster randomized controlled trial in rural Kerala, India
Source: PLoS One. 2018 Aug 22;13(8):e0201877. doi: 10.1371/journal.pone.0201877 (PMC6104953; doi:10.1371/journal.pone.0201877)

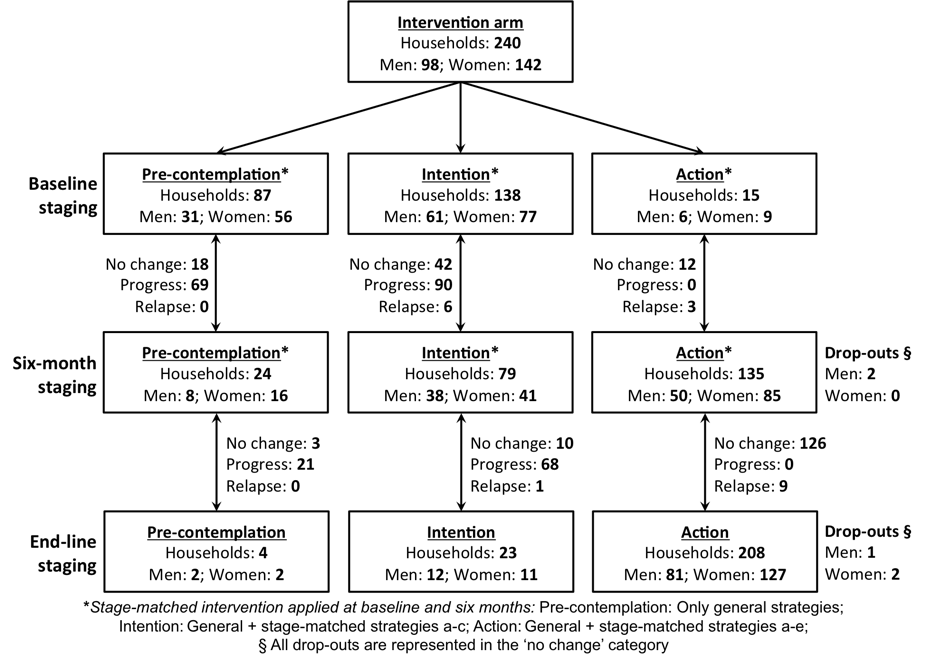

Supplement: S1 Fig — (TIFF) [file pone.0201877.s004.tiff]
